# Bogijetong Decoction and Its Selected Formulation Are Involved in Alleviating Neuropathic Pain in a Rat Model of Chronic Constrictive Injury

**DOI:** 10.1155/2018/2050636

**Published:** 2018-12-05

**Authors:** Ki-Joong Kim, Hye-Jeong Ahn, Uk Namgung, Chung Sik Cho

**Affiliations:** Department of Korean Medicine, Daejeon University, Daejeon 300-716, Republic of Korea

## Abstract

Bogijetong decoction (BGJTD) is a formulation that is used for the treatment of neuropathic pain caused by cancer therapy, diabetes, and peripheral nerve injury. In the previous study, we selected four herbal constituents from BGJTD, formulated new decoction (BeD), and demonstrated its efficacy on the neuroprotection of peripheral sciatic nerve in streptozotocin-induced diabetic animals. Here, we report attenuating effects of BGJTD and BeD on neuropathic pain. Neuropathic pain was induced by ligation of the sciatic nerve to generate chronic constrictive injury (CCI). BeD was more effective than BGJTD in alleviating neuropathic pain lasting 3 – 4 weeks after CCI. In vivo administration of BeD did not alter the levels of brain-derived neurotrophic factor (BDNF) which were strongly induced by CCI in the sciatic nerve but downregulated TrkB production in the sciatic nerve. Downregulation of TrkB signals by BeD was confirmed in cultured DRG neurons. BGJTD was more effective in attenuating TNF-*α* production in the sciatic nerve than BeD, whereas BeD increased IL-6 more efficiently than BGJTD. Furthermore, phopsho-Erk1/2 was increased in the sciatic nerve and dorsal root ganglia (DRG) after BeD treatment. Neurite outgrowth of primary DRG neurons prepared from rats which had undergone CCI for 7 days was significantly increased in BeD-treated group of animals compared to the control and BGJTD-treated groups. Compositional comparison of BeD revealed that the neurite outgrowth was facilitated by the treatments of* Panax ginseng* and* Paeonia lactiflora*. Together, these data suggest that BeD induces unique molecular response at the injury site and may trigger retrograde signaling into the neuronal cell body to modulate pain responses.

## 1. Introduction

Neuropathy is peripheral nerve malady caused by diabetes, cancer, physical nerve damage, and mental illness such as depression, nicotine addiction, obesity, and stress. Clinically, neuropathic pain is considered as primary target for the treatment of neuropathy because this pain is very severe and persistent. Although A*δ* and c-fibers are peripheral mediators of neuropathic pain, ascending spinal nerves and associated peripheral Schwann cells and spinal microglial cells are closely related to mediating pain transmission to the brain [1]. Chemical mediators such as fractalkine, substance P, ATP, and others that are secreted from these cells are known to be involved in the transmission of pain signals.

Neuropathic pain is chronic and persistent and lowers the quality of life. Neuropathic pain is not only controlled by pain-transmitting pathway but also affected by autonomic, endocrine, and immune influences [1, 2]. Currently, surgical procedures that regulate spinal tracts and several drugs including antidepressants and anticonvulsants are used to alleviate the severity of pain, but the therapeutic approaches curing neuropathic pain are not available. Several lines of studies using experimental animals provide evidence that molecular factors that are produced and released from nerve fibers and glial cells are involved in pathological responsiveness of neuropathic pain. For instance, nerve growth factor (NGF) and brain-derived neurotrophic factor (BDNF) produced from Schwann cells and spinal microglia cells and astrocytes are involved in generating pain signals by activating TrkB receptors and low affinity p75 neurotrophin receptors (p75NTR) [3]. Fractalkine is produced from sensory neurons and induces chemotactic responses of microglial cells and consequent inflammatory responses [4]. Moreover, inflammatory cytokines such as leukocyte-inducing factor (LIF), IL-1*β*, TNF-*α*, and NF-kB are produced from peripheral and spinal glial cells and contribute to pain signaling. Tetracycline as a regulator of inflammatory cytokines and nitric oxide, propentofylline regulating reactive astrocytes, and AV411 that induces the production of anti-inflammatory cytokine IL-10 [5–7] are known to regulate pain, but their efficacy is highly variable thereby accompanied with some side effects.

BGJTD is herbal formulation composed of 18 different herbal components. Based on traditional Korean medical theory, BGJTD was developed and used for the treatment of neuropathic pain accompanied as a sequela of cancer and diabetes [8, 9]. In order to understand biological basis of the action of BGJTD, previously we examined the effects of BGJTD on axonal regeneration after peripheral nerve injury and identified molecular factors associated with its efficacy on nerve regeneration [10]. To further characterize active herbal components among 18 different herbal components, we classified BGJTD into four subgroups BGJTD_a_ to BGJTD_d_ of herbal formulation based on traditional medicinal theory and constituted a new decoction BGJTD_e_, shortly BeD, which showed comparable efficacy to BGJTD in inducing responsiveness of injured axons [11]. While these studies strongly suggest that BGJTD and BeD are involved in regulating neuropathic pain in the injured nerves and diabetic neuropathy, its direct effect on mechanically triggered neuropathic pain remains to be studied. Here in the present study, we explored the regulatory effects of BGJTD and BeD on neuropathic pain using an animal model of chronic constrictive injury (CCI) in which the peripheral sciatic nerve was ligated. Our data provide evidence that BeD alleviates neuropathic pain and is involved in regulating BDNF-TrkB and IL-6 signaling pathways in the injured neurons. Furthermore, compositional analysis of BeD on neurite outgrowth highlights the significance of specific herbal constituents of BeD in regulating neuropathic pain.

## 2. Materials and Methods

### 2.1. Herbal Drug Extraction

BGJTD is a mixture composed of 18 different herbal drugs:* Astragalus membranaceus, Panax ginseng, Angelica gigas, Rehmannia glutinosa, Cnidium officinale, Paeonia lactiflora, Salvia miltiorrhiza, Prunus persica, Carthamus tinctorius, Spatholobus suberectus, Epimedium koreanum, *Lumbricidae* Whole Jiangxi, Pueraria thunbergiana, Pteridium aquilinum, Albizia julibrissin, Uncaria rhynchophylla, Chaenomeles sinensis,* and* Crassostrea gigas* [11]. We classified these constituents into 4 subgroups based on traditional medical theory, examined their effects on regenerative responses in cultured neurons, and constructed a new formula BeD composed of* Panax ginseng* (PG; Ginseng Radix),* Crassostrea gigas* (CG),* Angelica gigas* (AG), and* Paeonia lactiflora* (PL). Preparations and administration of BGJTD and BeD were performed essentially as described in our previous studies [10, 11].

### 2.2. Animals

Sprague-Dawley rats (male, 200-250 g) and mice (albino ICR; 20-25 g) were purchased from Samtako (Seoul, Korea) and adjusted in animal rooms for 1 week before the use of experiment. Animal rooms were maintained at 22°C, 60% humidity, and a 12 h light and 12 h dark cycle. Animals were allowed to eat commercial chow (Samyang Co., Korea) and drink water ad libitum. Animal care and all experimental procedures were in accordance with the NIH guide for the care and use of laboratory animals and approved by the Committee on Use of Live Animals for Teaching and Research at Daejeon University (Daejeon, Korea).

### 2.3. Sciatic Nerve Surgeries

Rats and mice were anesthetized with ketamine (80 mg/kg) and xylazine (5 mg/kg). The sciatic nerve on the left leg was exposed from the middle thigh between the gluteus maximus muscle and biceps femoris muscle. For crush injury (CI), the exposed nerve was held tight with a pair of forceps for 30 s. For CCI in rats, the sciatic nerve exposed at the same area was penetrated with a sharp needle and a portion of 1/2 to 2/3 of the nerve was ligated tight with 5-0 and 7-0 nylon or silk threads (Ethicon, Somerville, NJ, USA). For CCI in mice, the sciatic nerve was exposed and ligation was performed similarly by using 7-0 nylon thread. Sham surgery was given on the area of the sciatic nerve by skin incision to expose the nerve and suture.

### 2.4. Western Blot Analysis

About 1 cm length of sciatic nerve and DRG at lumbar levels 4 and 5 were dissected and used to extract protein. Protein extraction, SDS-polyacryamide gel electrophoresis, blot transfer, and primary and secondary antibody reactions were performed as described previously [11]. Fifteen micrograms of protein were loaded to each lane of the gel to resolve protein. Primary antibodies used in the present study were anti-BDNF (1:1000, mouse, monoclonal, Abcam, Cambridge, MA, USA), anti-TrkB (1:800, Rabbit, polyclonal, Abcam), anti-GAP-43 (1:800, Rabbit, polyclonal, Abcam), anti-phopsho-Erk1/2 (1:1000, Rabbit, polyclonal, Cell Signaling, Danvers, MA), anti-IL-6 (1:800, mouse, monoclonal, Abcam), anti-TNF-*α* (1:800, Rabbit, polyclonal, Abcam), and anti-Actin (1:20000, mouse, Sigma) antibodies, and secondary antibodies were goat anti-mouse and goat anti-rabbit horseradish peroxidase- (HRP-) conjugated antibodies (1:1000, Cell Signaling). Quantification of protein band intensity was performed by using the i-Solution software (Image & Microscope Technology, Daejeon, Korea).

### 2.5. Immunofluorescence Staining

Sciatic nerve was dissected and embedded in mounting medium at -20°C. Longitudinal nerve sections (20 *μ*m) were cut by using cryostat (Leica, Wetzlar, Germany) and thaw-mounted on gelatin-coated slides. Pretreament, blocking, and reactions with primary and secondary antibodies were performed as described previously [12]. Primary antibodies used in this study were ainti-NF-200 antibody (1:800, monoclonal, Santa Cruz Biotech, Dallas, TX, USA), anti-NF-200 antibody (1:800, polyclonal, Santa Cruz Biotech), anti-BDNF antibody (1:400, polyclonal, Abcam), anti-TrkB antibody (1:400, monoclonal, Abcam), and anti-phospho-Erk1/2 antibody (1:400, polyclonal, Cell Signaling), and secondary antibodies were fluorescein-goat anti-mouse (1:400, Molecular Probes, Eugene, OR, USA) or rhodamine-goat anti-rabbit (1:400, Invitrogen, Carlsbad, NM, USA) secondary antibodies. Fluorescence images were observed by using fluorescence microscope (Nikon, Kona, Japan), captured, and analyzed by the Adobe Photoshop program.

### 2.6. Von Frey Tests

Behavioral measurement for pain response was performed by von Frey test. The animal was adjusted for 15 min by placing it on the platform of von Frey equipment. The foot of the hind leg was stimulated by filaments and animal's withdrawal reflex to stimulation was measured by scoring the number of reflexes out of 20 stimuli per each test. The mean value of paw withdrawal frequency for each animal was determined by three independent measurements which were conducted with a 30 min interval between each measurement. We measured paw withdrawal frequency, as an experimental procedure measuring pain responses to mechanical hyperalgesia [13, 14].

### 2.7. DRG Neuron Culture

Rat's sciatic nerves were exposed and subjected to either CI or CCI. DRG at lumbar levels 4 and 5 were dissected and used for primary neuron culture. Neuronal culture was performed as described previously [12]. Dissociated neurons (1.5 x 10^2^ cell) were plated on 12 mm coverslips in 24-well plate. Cells were cultured for 24 h and then treated with herbal drugs (0.5 mg/ml) for 24 hr. After fixation, cells were used for immunofluorescence staining as described above.

### 2.8. Statistical Analysis

Data were presented as mean ± standard error of mean (SEM). The mean number data among individual groups were compared by the one-way ANOVA followed by Tukey test post-hoc analysis (SPSS computer software version 21.0), and statistically significant differences were reported as p<0.05, p<0.01, and p<0.001.

## 3. Results

**Figure 1:**
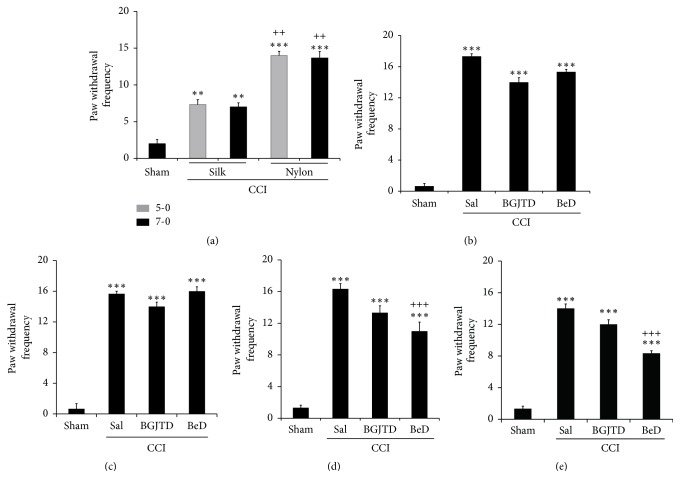
Changes in pain susceptibility in CCI animals by BGJTD and BeD administration. (a) Comparison of pain scores in CCI rats sutured with silk and nylon threads. In each thread, 5-0 and 7-0 sizes were examined. (b-e) Effects of CCI and drug (BGJTD, BeD) administration on pain scores in mice. One week after nerve ligation, animals showing pain scores higher than 15 in terms of paw withdrawal frequency in von Frey test were selected and randomly assigned into different animal groups and administered BGJTD, BeD, and saline (SAL) for 1 week (b), 2 weeks (c), 3 weeks (d), and 4 weeks (e). Number of animals in each group in (a) through (e) = 5. Error bar (mean ± SEM). *∗∗*p < 0.01 and *∗∗∗*p < 0.001 vs. sham controls. ^++^p<0.01 vs. CCI with corresponding silk threads in (a). ^+++^p<0.001 vs. CCI+SAL group in (d, e).

To determine the optimal condition inducing neuropathic pain in rats, we ligated sciatic nerves using two types of sutures and compared pain responses. Behavioral measurement of pain in terms of paw withdrawal frequency revealed that nylon suture was more efficient than silk suture (Figure 1(a)) and thus the nerve ligation in rats was performed using 5-0 nylon thread for the rest of the histobiochemical experiments in the present study. To determine the effects of BGJTD and BeD on behavioral pain responses, we administered BGJTD or BeD for 1 – 4 weeks into mice whose sciatic nerves had been ligated for 1 week. Pain susceptibility was not different between BGJTD- and BeD-injected animals after drug administration for 1 or 2 weeks (Figures 1(b) and 1(c)), but the response was significantly improved in animal group administered with BeD for 3 or 4 weeks compared with CCI+SAL group (Figures 1(d) and 1(e)). Pain susceptibility was also improved by BGJTD treatment but was less effective than BeD and statistically insignificant compared with CCI-SAL group.

**Figure 2:**
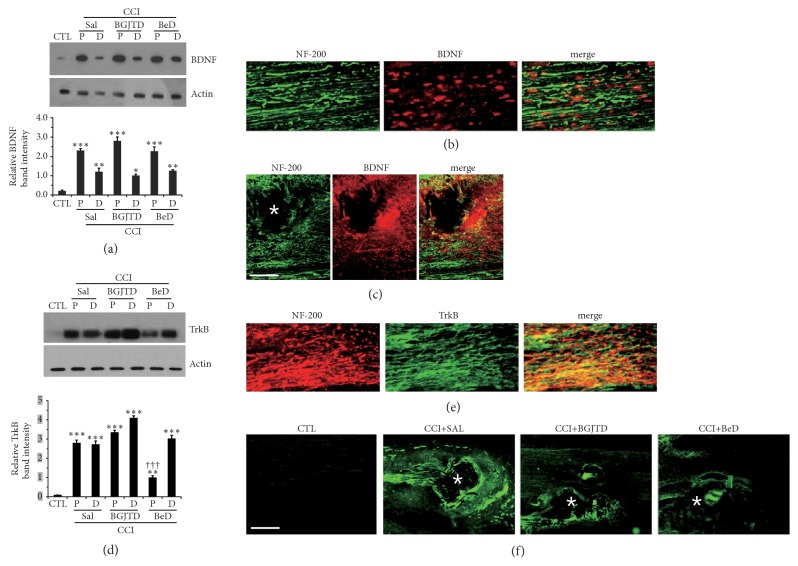
Effects of CCI and BGJTD/BeD administration on the production of BDNF and TrkB. (a) Western blot analysis of BDNF in the sciatic nerve. (b, c) Immunofluorescence analysis of BDNF signals in the sciatic nerve. In (b) and (c), longitudinal nerve sections were prepared from CCI+SAL animals. (d) Western blot analysis of TrkB in the sciatic nerve. (e, f) Immunofluorescence staining of TrkB signals in the sciatic nerves. Immunofluorescence images in (e) and (f) were from the longitudinal nerve sections of CCI+SAL animal. In (a) and (d), proximal (P) and distal (D) portions of the nerve were prepared and used for western blotting. Western blotting for actin was used as an internal loading control. Bar graphs (lower panel in (a) and (d)) represent quantification of relative band intensity to actin. Error bar (mean ± SEM, n=4). *∗*p<0.05, *∗∗*p<0.01, *∗∗∗*p<0.001 vs. control group. ^+++^p<0.001 vs. corresponding CCI+SAL group. In (c) and (f), areas of suture were marked by asterisks. Scale bar = 100 *μ*m.

The abovementioned data indicated that prolonged administration of BeD was effective for pain regulation. To investigate possible molecular factors related to the regulation of neuropathic pain by BGJTD and BeD, we prepared rat sciatic nerve which had been subjected to ligation for 1 week following 2 weeks of drug administration and analyzed proteins in the nerve. Levels of BDNF were significantly increased in the nerve after ligation particularly in the proximal portion and remained at similar levels after the treatment of both BGJTD and BeD (Figure 2(a)). BDNF signals were clearly observed in the nerve after ligation (Figure 2(b)), and particularly intense signals were detected around the area of nerve ligation (Figure 2(c)). We also found that TrkB protein, a major neuronal receptor of BDNF, was strongly induced in the nerve after ligation (Figure 2(d)). TrkB level was not altered by BGJTD treatment but significantly decreased in the proximal nerve by BeD treatment (Figure 2(d)). TrkB signals were mostly colocalized with NF-200 signals (Figure 2(e)). They were also seen strongly around the area of nerve ligation, which was largely attenuated by treatments of BeD (Figure 2(f)).

**Figure 3:**
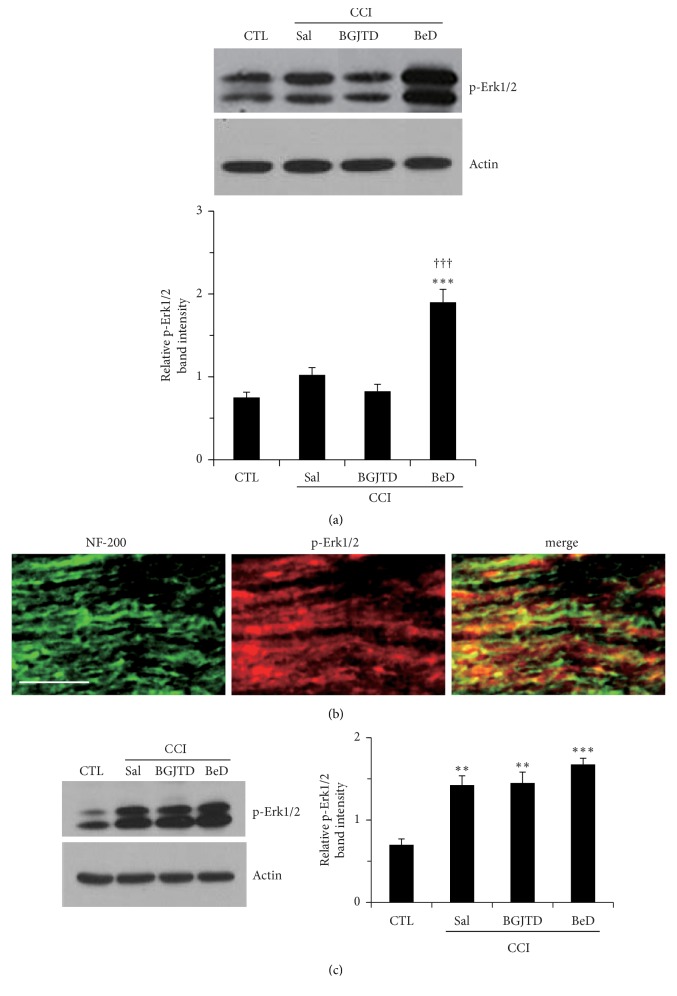
Regulation of Erk1/2 activation in the sciatic nerve and DRG after CCI and BGJTD/BeD treatments. (a, c) Western blot analysis of phospho-Erk1/2 in the sciatic nerve (a) and in the DRG (c). (b) Immunofluorescence images of phospho-Erk1/2 in the sciatic nerve. These are the representative images from an animal treated with CCI+BeD. Western blotting for actin was used as an internal loading control. Bar graphs in (a) and (c) represent quantification of relative band intensity to actin. Error bar (mean ± SEM, n=4). *∗∗*p<0.01, *∗∗∗*p<0.001 vs. control group. ^+++^p<0.001 vs. CCI+SAL group. Scale bar = 100 *μ*m.

Previous studies have well demonstrated that Erk1/2 protein is activated during the process of nerve repair after peripheral nerve injury [15]. Given that Erk1/2 activity is involved in TrkB signaling in neurons [16], we analyzed phospho-Erk1/2 in an area of nerve ligation and also in DRG neurons at lumbar levels 4 and 5 as retrograde target of sciatic sensory axons. There were some basal levels of phospho-Erk1/2 in both the nerve and DRG. Phospho-Erk1/2 was slightly elevated in the nerve after ligation and significantly increased by BeD (Figure 3(a)). Immunofluorescence staining for the nerve sections from BeD-treated CCI animals showed that while some of phospho-Erk1/2 signals were colocalized with NF-200-stained axons, others were not, suggesting their production from nonneuronal cells (Figure 3(b)). Phospho-Erk1/2 level was significantly increased in the DRG after nerve ligation and further upregulated by BeD treatment (Figure 3(c)).

**Figure 4:**
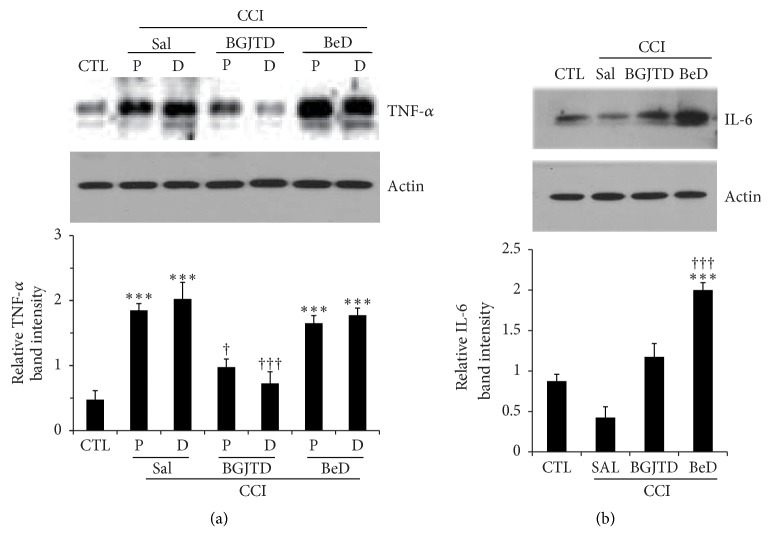
Comparison of TNF-*α* and IL-6 production in the sciatic nerve after CCI and BGJTD/BeD treatments. Western blot analysis of TNF-*α* (a) and IL-6 (b) in the proximal (P) and distal (D) stumps of the sciatic nerve. Bar graphs (lower panel in (a) and (b)) represent quantification of relative band intensity to actin. Error bar (mean ± SEM, n=4). *∗∗∗*p<0.001 vs. control group. ^+^p<0.05, ^+++^p<0.001 vs. corresponding CCI+SAL group.

To examine whether BGJTD/BeD administration affected inflammatory responses in association with neuropathic pain, we analyzed inflammatory cytokines TNF-*α* and IL-6 in the nerve. TNF-*α* levels were significantly increased after nerve ligation and then decreased by BGJTD treatment, but not by BeD (Figure 4(a)). Interestingly, however, levels of IL-6 did not show significant changes after CCI and BGJTD treatment but were greatly elevated by BeD treatment (Figure 4(b)).

**Figure 5:**
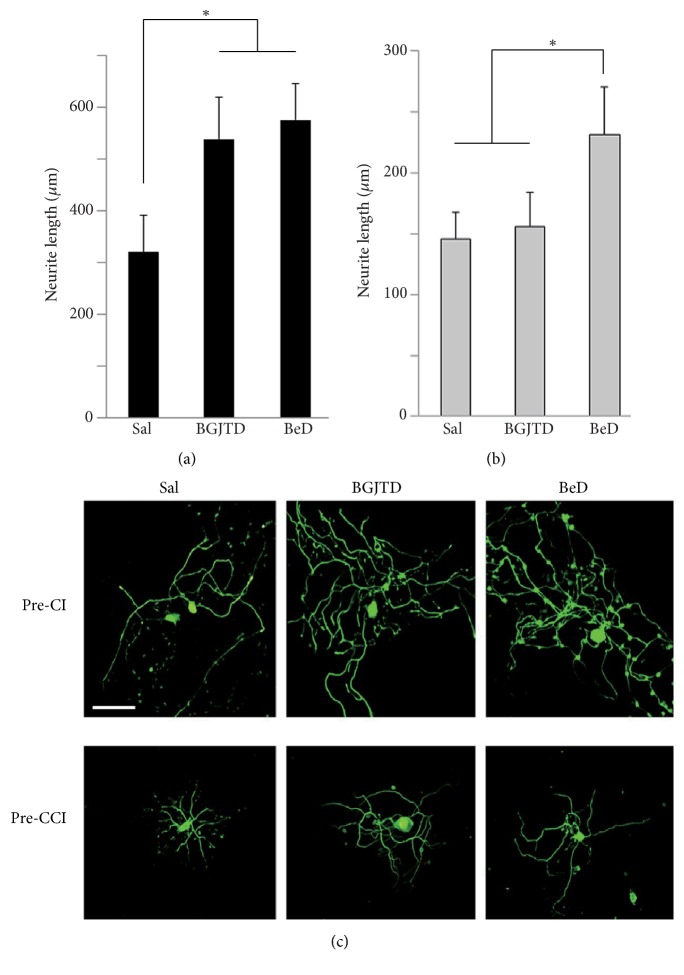
Effects of BGJTD and BeD treatment on the neurite outgrowth in cultured DRG neurons. Primary DRG neurons were prepared from rats which had undergone preconditioning CI (Pre-CI) in the sciatic nerve for 7 days (a) or preconditioning CCI (Pre-CCI) for 7 days (b). (c) Representative immunofluorescence images. Cultured neurons were visualized by green fluorescence of NF-200 protein. Bar plots denote mean ± SEM (n = 4). *∗*p < 0.05. Scale bar=100 *μ*m.

**Figure 6:**
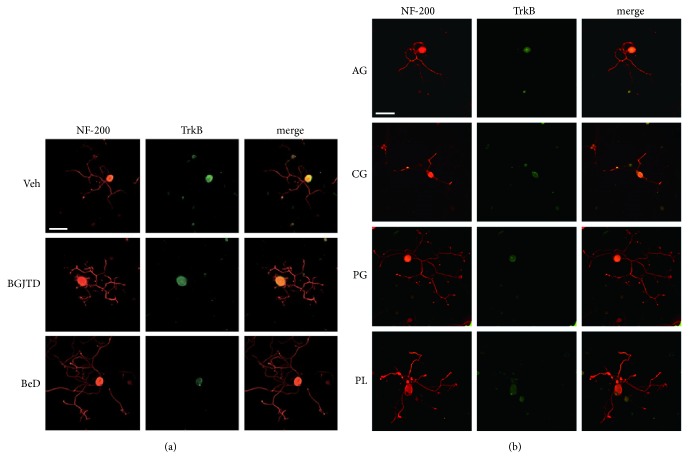
Immunofluorescence analysis of TrkB signals in cultured DRG neurons. Primary DRG neurons were prepared from rats given CCI in the sciatic nerve for 7 days. Neurons were treated with BGJTD and BeD (a) or individual herbal constituents of BeD (b) for 24 h prior to fixation for immunofluorescence staining for NF-200 (red) and TrkB (green) as indicated in the figure. Scale bar = 100 *μ*m.

It is well known that preconditioning injury (e.g., crush injury on the sciatic nerve) enhances neurite outgrowth of DRG sensory neurons in culture [17]. Here, we investigated the effect of BGJTD/BeD on the neurite outgrowth of DRG neurons in rats which had undergone CI or CCI in the sciatic nerve for 7 days. In DRG neurons prepared after CI, treatments of BGJTD and BeD significantly increased neurite outgrowth (Figure 5(a)). Neurite outgrowth was notably reduced in DRG neurons prepared after CCI, compared with those after CI (see Figures 5(a) and 5(b)). Treatment of BeD, but not BGJTD, significantly enhanced neurite outgrowth (Figure 5(b)). Representative images of DRG neurons are shown in Figure 5(c). Using cultured DRG neurons which were prepared 1 week after nerve ligation, we further analyzed immunofluorescence signals of TrkB protein. TrkB signals were clearly observed in neurons treated with saline vehicle and with BGJTD as well, but the signal intensity was clearly diminished after BeD treatment (Figure 6(a)). To further examine which herbal constituents were effective in regulating neurite outgrowth and TrkB expression, we treated cells with individual herbal components of BeD. Neurite outgrowth, as visualized by NF-200-staining of neurons, was prominent in cells treated with PG and PL (Figure 6(b)). Overall TrkB signal intensity in cell groups treated with 4 different herbal constituents was weaker than those of vehicle control group.

## 4. Discussion

In the previous study, we demonstrated that BGJTD and BeD had a protective effect on neuropathic insults in streptozotocin-induced diabetic animals [11]. Here, using an animal model of CCI, we investigated the effects of BGJTD and BeD on peripheral neuropathy. BeD administration alleviated pain response in CCI animals and changed the production levels of nerve factors that are related to pathologic responsiveness of pain. BGJTD was partially effective in alleviating pain. BeD appears to be more effective than BGJTD in regulating the production of TrkB, Erk1/2, and inflammatory cytokine IL-6, raising an issue that it is important to select efficacious components in formulating herbal decoctions while excluding some inefficacious herbal components.

**Figure 7:**
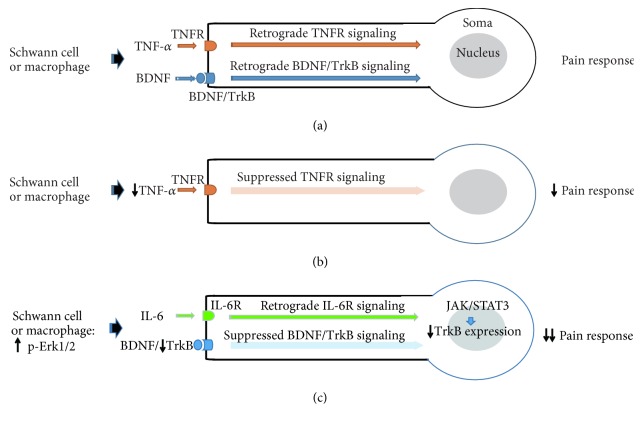
Schematics of proposed mechanisms underlying BGJTD- or BeD-modulated pain responses. According to our data, signaling of TNF-*α* and TrkB appeared to increase after nerve ligation (a). TNF-*α*, which were decreased by BGJTD treatment, may cause weakening in retrograde signaling of TNFR into the cell body, leading to alleviating pain response (b). After BeD treatment, p-Erk1/2 and IL-6 signals, induced from Schwann cells and macrophages, may transmit the signals of IL-6R into the neuronal cell body (soma), inducing JAK/STAT activation and regulation of target gene expression including the repression of TrkB gene expression (c). Consequently, retrograde TrkB signaling would be attenuated and lead to alleviate pain response.

Peripheral nerve ligation is an experimental procedure that is widely used to induce and examine neuropathic pain. We found that pain response in the hind limb was clearly induced after nerve ligation and significantly attenuated by prolonged administration of BeD lasting 3-4 weeks. We assumed that possible biochemical events, if any, might occur in less than 3 weeks before the behavioral consequences and thus investigated molecular factors that could be involved in pathologic responsiveness in the injured nerve. In the peripheral nerve, BDNF is induced at gene expression levels in neurons and nonneuronal cells such as Schwann cells and transmits signals by interacting with neuronal receptors such as TrkB and p75 in an autocrine or paracrine manner [18]. BDNF signaling through TrkB is known to augment pain response in the peripheral nerves and spinal cord after injury [19–21]. Consistent with previous reports, BDNF and TrkB proteins were strongly induced in the sciatic nerve after CCI. Some BDNF signals were not colocalized with NF-200-stained nerve fibers, indicating its expression from Schwann cells and macrophages, which are activated after nerve ligation. Together, locally induced BDNF would be bound to TrkB, entrapped into the vesicles, and retrogradely transported into the cell body to induce target gene expression [22]. In addition, our data indicate that TNF-*α*, which was upregulated in the injured nerve, may transmit signals to axons via TNF receptor (TNFR). TNFR may activate intracellular signaling pathways leading to target gene expression and inflammatory responses [23, 24] (see Figure 7(a)).

Then, the administration of BGJTD and BeD resulted in a characteristic pattern of the production of nerve proteins. Treatment of BGJTD in CCI animals did not change the production of BDNF, TrkB, and phospho-Erk1/2 but largely decreased TNF-*α* level. TNF-*α* is produced from Schwann cells and macrophages in the peripheral nerves after CCI, binds TNFR, and transmits inflammatory signals into the cell body [23, 25]. Therefore, BGJTD may exert the alleviating effect on pain by inhibiting the retrograde transmission of TNF-*α*/TNFR signaling into the cell body (Figure 7(b)).

Unlike BGJTD, treatment of BeD did not alter the level of TNF-*α* but increased IL-6 in the injured nerve area of CCI animals. Moreover, levels of phospho-Erk1/2 and TrkB proteins were significantly elevated in the injured nerve. Previously, we reported that Erk1/2 facilitated peripheral nerve regeneration by activating Schwann cells [15], and other studies reported that Erk1/2 activity, which is induced at the injury site of the peripheral nerve, is involved in retrogradely transporting the lesion signal into the cell body [26, 27]. Given the notion that a certain portion of phospho-Erk1/2 signals are found in nonaxonal area (Figure 3(b)), we speculate that BeD may upregulate Erk1/2 level in Schwann cells and macrophages, as has been reported previously. Previous study also demonstrated that Erk1/2 activation is related to an increase of IL-6 synthesis via the activation of NF-kB in astrocytes [28]. IL-6 is known to act as both proinflammatory and anti-inflammatory cytokines [29]. For instance, IL-6 produced from Schwann cells after nerve injury functions to support axonal regeneration and neuroprotection and alleviates neuropathic pain [30, 31]. Here, IL-6 level was not increased by CCI, and thus IL-6 certainly did not act as proinflammatory cytokine at least in the peripheral nerve undergoing neuropathy. Then, BeD treatment strongly elevated IL-6 level, suggesting the augmentation of anti-inflammatory response possibly mediated by IL-6. How would IL-6 produced from Schwann cells and macrophages in BeD-treated CCI animals communicate with injured axons and modulate neuropathic pain? There are IL-6 receptors in peripheral axons known to transmit signals into JAK/STAT3 signaling molecules in the neuronal cell body [32, 33] and induce target gene expression at transcriptional level. TrkB was induced in cultured DRG neurons which were prepared from animals given preconditioning CCI but attenuated by BeD treatment. We speculate that retrograde signals from IL-6 receptors may be involved in inhibiting TrkB gene expression, which results in reduced TrkB level at the injured nerve area, further leading to a large reduction of pain response (Figure 7(c)). While further study is critical to verify the abovementioned proposal, it seems clear that molecular mechanisms underlying the regulatory effects of BGJTD and BeD on peripheral neuropathy are different.

Primary DRG neurons can be prepared from adult animals in which preconditioning injury had been given, and their neurite outgrowth is widely used to analyze regenerative responses of injured axons [17, 34]. Using a similar experimental paradigm, we prepared primary DRG neurons after CCI and compared their neurite outgrowth with that of DRG neurons prepared after CI. Neurite outgrowth of neurons from CCI animals was much shorter than those from CI animals. This difference could be partly due to the fact that putative regeneration-inducing signals that might be produced from the distal stump of the injured nerve cannot pass through the constrictive axons. Yet, BeD treatment promoted neurite outgrowth, implying the potential role of BeD for the repair process of peripheral axons after ligation injury. When the cells were treated with individual herbal constituents of BeD, PG and PL were more effective in enhancing neurite outgrowth than AG and CG. However, all four herbal constituents of BeD similarly attenuated TrkB signals in cultured cells, suggesting that the neuronal responses to individual herbal constituents, in terms of neurite outgrowth and TrkB signaling, were partially, but not completely, correlated to each other. Further study is required to clarify how the intrinsic neuronal ability of axonal regeneration is linked to the regulation of neuropathic pain when BeD and BGJTD were administered for therapeutic purpose.

## 5. Conclusion

Our data suggest that BNDF-TrkB signal pathway may be activated within the nerve fibers after CCI and contribute to the transmission of neuropathic pain signals into neuronal cell body. Administration of BeD may lead to downregulating TrkB levels in axons and attenuating the transmission of pain signals. Alternatively, BeD may directly act on sensory neurons in DRG or even spinal glial cells and affect pain susceptibility because BeD was systemically administered [1]. Examination of pain response after focal injection of BeD into the area of nerve ligation may help clarify this issue. It is unclear at this moment why BGJTD was not effective for the regulation of BDNF-TrkB pathway; the effect mediated by negatively acting herbal components that are present only in BGJTD may be considered.

In conclusion, our study highlights that a reconstituted formulation BeD may be able to produce an effect for the regulation of neuropathic pain comparable with or even superior to the original BGJTD. The experimental approach shown here provides insights into the methodological basis for compositional analysis of multiple herbal constituents of the decoction.

## Data Availability

The data used to support the findings of this study are available from the corresponding author upon request.

## Abbreviations

BGJTD: Bogijetong decoction
BeD: Bogijetong_e_ decoction
CCI: Chronic constrictive injury
CI: Crush injury
DRG: Dorsal root ganglion
BDNF: Brain-derived neurotrophic factor.
